# Supplementary material for: Pyruvate Dehydrogenase Kinase 1 inhibition mediated oxidative phosphorylation enhancement in cartilage promotes osteoarthritis progression
Source: BMC Musculoskelet Disord. 2023 Jul 20;24:597. doi: 10.1186/s12891-023-06585-6 (PMC10357736; doi:10.1186/s12891-023-06585-6)
Supplement: Supplementary file 1 — Additional file 1. [file 12891_2023_6585_MOESM1_ESM.zip › BP.Gsea.1653623667859/GOBP_CELLULAR_RESPONSE_TO_ARSENIC_CONTAINING_SUBSTANCE.html]

Details for gene set GOBP\_CELLULAR\_RESPONSE\_TO\_ARSENIC\_CONTAINING\_SUBSTANCE[GSEA]

|  || Dataset | 55457\_collapsed\_to\_symbols.cls.cls #OA\_versus\_normal.cls.cls #OA\_versus\_normal\_repos |
| Phenotype | cls.cls#OA\_versus\_normal\_repos |
| Upregulated in class | normal |
| GeneSet | GOBP\_CELLULAR\_RESPONSE\_TO\_ARSENIC\_CONTAINING\_SUBSTANCE |
| Enrichment Score (ES) | -0.5948036 |
| Normalized Enrichment Score (NES) | -1.7247823 |
| Nominal p-value | 0.0050083473 |
| FDR q-value | 0.12095836 |
| FWER p-Value | 1.0 |
Table: GSEA Results Summary

  

Fig 1: Enrichment plot: GOBP\_CELLULAR\_RESPONSE\_TO\_ARSENIC\_CONTAINING\_SUBSTANCE      
 Profile of the Running ES Score & Positions of GeneSet Members on the Rank Ordered List

  

| SYMBOL | TITLE | RANK IN GENE LIST | RANK METRIC SCORE | RUNNING ES | CORE ENRICHMENT || 1 | UROS | uroporphyrinogen III synthase [Source:HGNC Symbol;Acc:HGNC:12592] | 323 | 0.735 | 0.0585 | No |
| 2 | ZFAND1 | zinc finger AN1-type containing 1 [Source:HGNC Symbol;Acc:HGNC:25858] | 1984 | 0.363 | -0.0292 | No |
| 3 | MAPK13 | mitogen-activated protein kinase 13 [Source:HGNC Symbol;Acc:HGNC:6875] | 2544 | 0.298 | -0.0387 | No |
| 4 | VCP | valosin containing protein [Source:HGNC Symbol;Acc:HGNC:12666] | 3518 | 0.201 | -0.0914 | No |
| 5 | GSTO1 | glutathione S-transferase omega 1 [Source:HGNC Symbol;Acc:HGNC:13312] | 6275 | -0.025 | -0.3024 | No |
| 6 | DAXX | death domain associated protein [Source:HGNC Symbol;Acc:HGNC:2681] | 8926 | -0.226 | -0.4824 | No |
| 7 | ZC3H12A | zinc finger CCCH-type containing 12A [Source:HGNC Symbol;Acc:HGNC:26259] | 9030 | -0.234 | -0.4638 | No |
| 8 | PPIF | peptidylprolyl isomerase F [Source:HGNC Symbol;Acc:HGNC:9259] | 9999 | -0.321 | -0.5025 | No |
| 9 | HSF1 | heat shock transcription factor 1 [Source:HGNC Symbol;Acc:HGNC:5224] | 11189 | -0.456 | -0.5430 | Yes |
| 10 | SLC38A2 | solute carrier family 38 member 2 [Source:HGNC Symbol;Acc:HGNC:13448] | 11722 | -0.536 | -0.5234 | Yes |
| 11 | ATF4 | activating transcription factor 4 [Source:HGNC Symbol;Acc:HGNC:786] | 11999 | -0.593 | -0.4774 | Yes |
| 12 | MKNK2 | MAPK interacting serine/threonine kinase 2 [Source:HGNC Symbol;Acc:HGNC:7111] | 12236 | -0.655 | -0.4213 | Yes |
| 13 | HNRNPA1 | heterogeneous nuclear ribonucleoprotein A1 [Source:HGNC Symbol;Acc:HGNC:5031] | 12356 | -0.693 | -0.3518 | Yes |
| 14 | HMOX1 | heme oxygenase 1 [Source:HGNC Symbol;Acc:HGNC:5013] | 12421 | -0.714 | -0.2757 | Yes |
| 15 | ATF3 | activating transcription factor 3 [Source:HGNC Symbol;Acc:HGNC:785] | 12659 | -0.833 | -0.1995 | Yes |
| 16 | DDX3X | DEAD-box helicase 3 X-linked [Source:HGNC Symbol;Acc:HGNC:2745] | 12788 | -0.949 | -0.1015 | Yes |
| 17 | MYC | "MYC proto-oncogene, bHLH transcription factor [Source:HGNC Symbol;Acc:HGNC:7553]" | 12801 | -0.970 | 0.0077 | Yes |
Table: GSEA details [plain text format]

  

Fig 2: GOBP\_CELLULAR\_RESPONSE\_TO\_ARSENIC\_CONTAINING\_SUBSTANCE      
 Blue-Pink O' Gram in the Space of the Analyzed GeneSet

  

Fig 3: GOBP\_CELLULAR\_RESPONSE\_TO\_ARSENIC\_CONTAINING\_SUBSTANCE: Random ES distribution      
 Gene set null distribution of ES for **GOBP\_CELLULAR\_RESPONSE\_TO\_ARSENIC\_CONTAINING\_SUBSTANCE**
